# Psychological capital in cancer patients and family caregivers: determinants, measurement, outcomes, and intervention strategies

**DOI:** 10.3389/fpsyg.2026.1957751

**Published:** 2026-09-23

**Authors:** Yan Li, Vimala Ramoo, Xingling Zeng, Liu He, Juan Du, Yuhong Zhang, Xiaoya Liu, Xinjing Xiang

**Affiliations:** 1 Faculty of Health Sciences, Nursing, and Education, MAHSA University, Jenjarom, Selangor, Malaysia; 2 The Second Affiliated Hospital of Chengdu Medical College, Nuclear Industry Hospital, Chengdu, China; 3 School of Nursing, Jiangsu Food & Pharmaceutical Science College, Huai’an, Jiangsu, China

**Keywords:** cancer, dyadic coping, family caregivers, psychological capital, supportive oncology

## Abstract

**Background:**

As cancer care increasingly extends into outpatient, community and home settings, patients and family caregivers must manage prolonged uncertainty, treatment toxicity, financial strain and role disruption. Psychological capital (PsyCap), comprising self-efficacy, optimism, hope and resilience, offers a strengths-based framework for understanding adaptation in cancer care.

**Objective:**

To synthesize and conceptually integrate the literature on PsyCap in adult cancer patients, family caregivers and patient–caregiver dyads, with particular attention to determinants, measurement approaches, associated outcomes and intervention strategies relevant to supportive oncology.

**Methods:**

A structured narrative review was conducted to synthesize the literature on psychological capital (PsyCap) among cancer patients and family caregivers. A structured database search was performed in PubMed/MEDLINE, Web of Science, CINAHL and PsycINFO, supplemented by citation tracking. Publications addressing the conceptualization, determinants, measurement, outcomes and intervention strategies of PsyCap in cancer care were included. The findings were synthesized narratively using an evidence-mapping approach.

**Results:**

Seventy publications were included in the synthesis. The evidence suggests that composite PsyCap, as well as its individual component resources (resilience, hope, optimism, and self-efficacy), are associated with psychological adjustment, coping, self-management, and quality of life among cancer patients, while similar associations have been reported among caregivers. PsyCap appears to be influenced by multiple clinical and contextual factors, including symptom burden, treatment demands, uncertainty, financial stress, and caregiving responsibilities. Direct measurement of PsyCap as a higher-order construct remains relatively uncommon, and caregiver-focused and dyadic assessments are limited. Intervention studies indicate that mindfulness-based programs, positive psychological interventions informed by the PERMA model (Positive Emotion, Engagement, Relationships, Meaning, and Accomplishment), exercise and body–mind practices, and dyadic or family-based approaches may strengthen PsyCap-related resources and improve psychosocial outcomes. However, most interventions have not explicitly evaluated PsyCap as a primary outcome.

**Conclusion:**

PsyCap provides a useful framework for understanding positive psychological adaptation in cancer care. Existing evidence suggests that PsyCap may represent a modifiable target for supportive oncology interventions and may operate at both individual and relational levels. Future research should prioritize oncology-specific measurement approaches, longitudinal investigations, caregiver and dyadic research, and rigorously designed intervention studies that directly assess PsyCap outcomes.

## 1 Introduction

Cancer continues to be one of the world’s leading causes of illness and death, but the psychological impact of cancer is no longer restricted to diagnosis, treatment and survival rates (Siegel et al., 2023). The shift in cancer care from acute hospital-based to ambulatory, community and home-based care means patients and family caregivers must cope with the uncertainty, side effects, impairment, financial burden and care-coordinating demands (Lee et al., 2018; Manne and Badr, 2008; Weitkamp et al., 2021; Chua et al., 2020; Wang J. et al., 2024). Psychological effects of cancer are therefore not limited to the individual patient. Cancer tends to create a co-evolving environment of stress in which patients and families interact to influence one another’s emotions, coping processes, communication and quality of life (Manne and Badr, 2008; Weitkamp et al., 2021; Wang et al., 2021).

Classic psycho-oncology has been crucial in highlighting anxiety, depression, symptom distress, caregiver burden and needs. But a focus on deficits may overlook the positive psychological resources that help patients and families to stay engaged in cancer treatment, regain a sense of control and maintain meaning during times of uncertainty (Manne and Badr, 2008; Weitkamp et al., 2021; Wang et al., 2021). Psychological capital (PsyCap), a term first used in positive psychology and positive organizational behavior, is a helpful strengths-based framework. It is a composite of four resources: self-efficacy, optimism, hope and resilience (Luthans and Youssef, 2004; Luthans et al., 2005; Zhang et al., 2010; Luthans et al., 2006; Luthans et al., 2010). These resources are theoretically interrelated but clinically interactive. Self-efficacy provides confidence in managing symptoms and treatment; optimism provides realistic positive expectations; hope combines agency and pathway thinking to set and pursue goals; and resilience allows recovery from adversity.

Oncology is particularly concerned with PsyCap for three reasons. First, cancer and its treatment can challenge all four PsyCap components through symptom burden, body changes, fear of recurrence, treatment failure, financial toxicity, and disruptions to everyday roles and relationships (Sebri et al., 2021; Presley et al., 2024; Khan et al., 2023; Lin and Tsay, 2005; Feldman and Corn, 2023; Eala et al., 2022). Second, PsyCap is generally considered a dynamic psychological resource that can be strengthened through intervention, learning experiences, and supportive environments (Luthans et al., 2006; Luthans et al., 2010). Third, cancer is a relational experience that affects both patients and family caregivers. Psychological resources that constitute PsyCap, including hope, self-efficacy and resilience, may be shared within the dyad (Manne and Badr, 2008; Weitkamp et al., 2021; Wang et al., 2021; Zemp et al., 2017; Gao et al., 2025; von Heymann-Horan et al., 2019; Wang et al., 2025; Sun et al., 2025). Consistent with dyadic coping theory and family systems theory, PsyCap may therefore be understood as both an individual and a relational resource.

While there is a growing body of research, it is sparse. Research varies in terms of language, methods, cancer types, intervention types and outcomes. Some studies explore only one dimension (resilience, hope) rather than PsyCap as a higher-order construct. Alternatively, some interventions that are potentially effective for PsyCap are not reported as PsyCap interventions, so the evidence is also hard to integrate. Few caregiver-focused and dyadic studies have been conducted compared to patient-focused studies, and few studies have focused on testing mechanisms. Given these gaps, this review aims to provide a structured synthesis of the emerging literature on PsyCap in cancer care. Specifically, the review addresses four questions: (i) how PsyCap can be conceptualized in cancer settings; (ii) what is known about its determinants, measurement, and clinical relevance; (iii) which intervention strategies may contribute to its development; and (iv) what methodological and implementation priorities should guide future research.

## 2 Methods

### 2.1 Design

This review was designed as a structured narrative review to synthesize the literature on psychological capital (PsyCap) in cancer patients, family caregivers, and patient–caregiver dyads. This approach was chosen over a formal systematic review or scoping review because the considerable heterogeneity in study designs, populations, and outcome measures across the field precludes meaningful quantitative synthesis and makes strict methodological comparison inappropriate. The primary aim was to provide a broad, theory-informed narrative integration to identify patterns and gaps, rather than to answer a narrowly defined clinical question. A structured literature search and evidence-mapping approach was used to systematically identify and organize evidence across diverse study designs, with findings integrated through narrative and theory-informed synthesis rather than quantitative pooling. The review process did not involve independent duplicate screening or formal risk-of-bias assessment. Nevertheless, it is important to acknowledge that the lack of duplicate screening and quality appraisal means that the methodological rigor of individual included studies was not systematically assessed, which should be considered when interpreting the synthesized findings.

### 2.2 Data sources and search strategy

A structured literature search was conducted in PubMed/MEDLINE, Web of Science Core Collection, CINAHL, and PsycINFO. Citation tracking of relevant reviews and key intervention studies was subsequently undertaken to identify additional relevant publications that were not captured through the database searches. Purposive searching was used to identify foundational theoretical, conceptual, measurement, and methodological publications directly relevant to the review framework, including selected non-oncology literature. These publications were selected based on their direct relevance rather than citation counts or other formal ranking criteria. These complementary approaches served distinct purposes: database searching and citation tracking primarily identified oncology-related empirical and intervention literature, whereas purposive searching provided theoretical, conceptual, measurement, and methodological context.

The search strategy was developed around four concept domains: (1) cancer; (2) psychological capital and related psychological resources; (3) family caregiving and dyadic processes; and (4) psychosocial intervention approaches. Search terms were adapted to the indexing systems and interfaces of individual databases. Representative database search strategies are provided in Supplementary Table S1. Searches were conducted in January 2026 and covered publications from database inception to January 2026 and the search was not formally updated prior to manuscript submission. The search was intended to provide broad and structured coverage of the relevant literature rather than to constitute a formal systematic review search conducted under systematic-review methodology. Title and abstract screening and full-text assessment were conducted by one reviewer. Given the structured narrative design of the review, duplicate independent screening was not performed.

### 2.3 Eligibility criteria

Studies were eligible if they involved adult cancer patients, family caregivers, or patient-caregiver dyads. Eligible studies addressed PsyCap as a higher-order construct or examined one or more of its component resources (self-efficacy, optimism, hope, and resilience). We included studies examining the conceptual foundations, measurement approaches, determinants, outcomes, or intervention strategies related to PsyCap in cancer care. Eligible publications included empirical studies, systematic reviews, meta-analyses, scale-development studies, and major conceptual articles. Studies were excluded if their primary subject matter fell outside the scope of cancer-related psychological adaptation and they provided no substantive contribution to the conceptualization, measurement, determinants, outcomes, interventions, or contextual understanding of PsyCap or its component resources. Publications from outside oncology were not part of the structured database search but were purposively incorporated only when they contributed essential theoretical, conceptual, or methodological insights relevant to understanding PsyCap in cancer care. The search was limited to publications available in English and to peer-reviewed journal articles. Grey literature, dissertations, conference proceedings, and non-peer-reviewed publications were not included.

### 2.4 Study selection and evidence mapping

Titles, abstracts, and full texts were reviewed for relevance to the review objectives. Information extracted from the included publications included author and year, study population, design, intervention or exposure, outcome domains, key findings, and relevance to PsyCap in cancer care. Publications contributing to the narrative synthesis were subsequently organized using an evidence-mapping approach to facilitate thematic synthesis rather than methodological comparison. Evidence was grouped according to its primary contribution to the review objectives into five domains: (1) conceptual foundations; (2) measurement approaches; (3) determinants and influencing factors; (4) patient, caregiver and dyadic outcomes; (5) intervention strategies. The first author conducted the coding and categorization using these predefined domains. Publications addressing more than one domain were categorized according to their primary contribution to the review objectives. The coding was conducted by the first author using the predefined five-domain framework, and publications addressing multiple domains were assigned according to their primary contribution to the review objectives. Because coding was performed by a single reviewer, formal inter-rater reliability assessment was not undertaken. The evidence-mapping framework used to organize the reviewed literature is summarized in Table 1 and guided the thematic organization of the subsequent Results and Discussion sections.

**Table 1:** Evidence Mapping Framework used to organize the reviewed literature.

Evidence domain	Purpose in the review	Examples of evidence
Conceptual foundations	Define PsyCap and theoretical basis	PsyCap theory, positive psychology, family systems, dyadic coping
Measurement approaches	Measurement and psychometrics	PCQ, oncology-specific scales
Determinants and influencing factors	Factors influencing PsyCap	Symptoms, uncertainty, caregiving burden, financial toxicity
Patient, caregiver and dyadic outcomes	Associations with adaptation	Quality of life, distress, coping, communication, resilience
Intervention strategies	Approaches that may strengthen PsyCap	Mindfulness, PERMA, exercise, dyadic interventions

## 3 Results

### 3.1 Overview of the evidence base

The narrative synthesis was informed by publications identified through structured database searching, citation tracking, and purposive searching. Collectively, these publications contributed evidence relating to the conceptualization of PsyCap, measurement approaches, determinants, psychosocial outcomes, and intervention strategies relevant to supportive oncology.

The reviewed literature included empirical studies, systematic reviews, meta-analyses, psychometric investigations, intervention evaluations, and conceptual papers. Publication activity increased substantially after 2020, reflecting growing interest in positive psychological resources in cancer care. Compared with studies involving patients, considerably fewer investigations focused specifically on family caregivers or patient–caregiver dyads.

The 70 publications were identified through the complementary search approaches described above. Because publication-level search provenance was not prospectively recorded, mutually exclusive counts for the individual search approaches could not be reliably reconstructed and are therefore not reported. The three search approaches contributed distinct types of evidence to the synthesis. The structured database searches yielded the majority of empirical studies examining PsyCap and its component resources in oncology populations. Citation tracking supplemented this with additional intervention evaluations and longitudinal studies not captured by the primary searches. Purposive searching provided the theoretical, conceptual, and methodological foundations that informed the review framework, including selected non-oncology literature on PsyCap theory and measurement. As publication-level search provenance was not prospectively recorded, mutually exclusive source counts are not reported; however, the complementary contributions of each approach are reflected in the thematic organization of the evidence presented below.

The 70 publications represented a heterogeneous body of evidence comprising 18 cross-sectional observational studies, including dyadic studies (25.7%); 17 intervention or experimental publications (24.3%); 14 systematic reviews or meta-analyses (20.0%); 11 conceptual, theoretical, or narrative publications (15.7%); 4 psychometric or instrument-development and validation studies (5.7%); 4 longitudinal observational studies (5.7%); 1 retrospective observational study (1.4%); and 1 epidemiological or descriptive report (1.4%). Given this heterogeneity, the publications were not treated as equivalent forms of evidence; rather, their study design, publication type, and contribution to the relevant evidence domain were considered when interpreting and synthesizing the findings.

An important distinction emerged regarding how psychological capital was represented across the included literature. Only a subset of publications explicitly examined PsyCap as a composite or higher-order construct, whereas others focused on individual PsyCap components or provided conceptual, contextual, dyadic, caregiver-related, intervention, or methodological evidence relevant to PsyCap. The distribution of publications according to their primary PsyCap focus is presented in Table 2.

**Table 2:** Distribution of included publications by primary PsyCap focus.

Primary PsyCap focus	Operational definition	*n*	%
Composite PsyCap	Publications that explicitly measured, analyzed, developed, or targeted psychological capital as an overall or higher-order construct	15	21.4
Individual PsyCap component(s)	Publications primarily examining resilience, hope, optimism, and/or self-efficacy without directly assessing PsyCap as an overall construct	10	14.3
Conceptual PsyCap literature	Publications primarily providing conceptual or theoretical foundations for PsyCap without directly measuring the construct	2	2.9
Other PsyCap-relevant supporting literature	Publications informing the cancer, caregiver, dyadic, intervention, positive psychology, or methodological context without directly measuring composite PsyCap or an individual component as the primary construct	43	61.4
Total		70	100.0

Categories were mutually exclusive and assigned according to the primary focus of each publication. Composite PsyCap refers to the explicit operationalization of psychological capital as an overall or higher-order construct. Individual component-focused publications examined one or more of the four theoretically defined PsyCap resources—self-efficacy, optimism, hope, or resilience—without directly assessing composite PsyCap.

The evidence identified across the included literature was subsequently integrated into an author-developed conceptual framework (Figure 1), which synthesizes the relationships among cancer-related stressors, psychological capital, patient–caregiver relational processes, psychosocial interventions, and adaptive outcomes.

**Figure 1:**
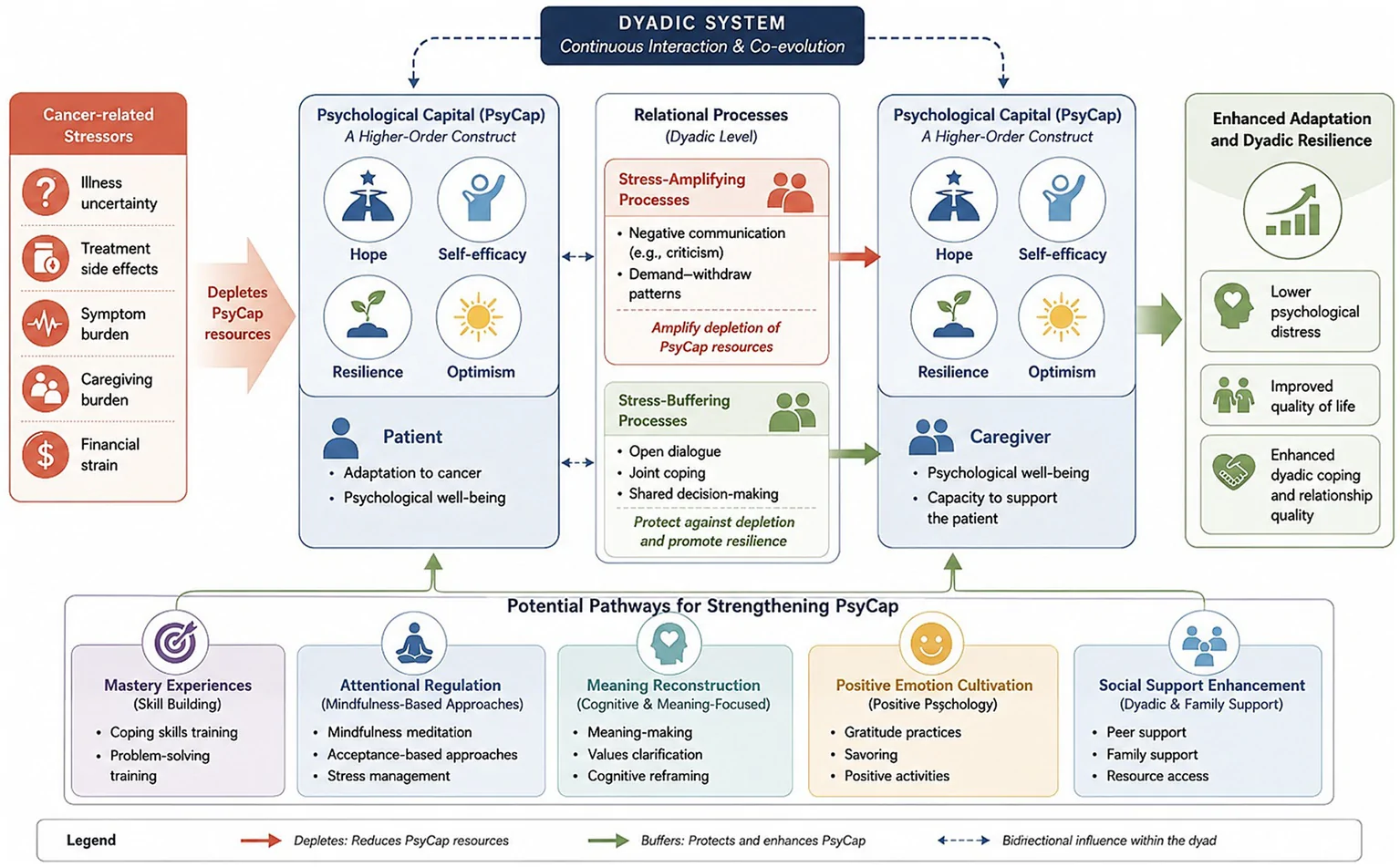
Hypothesis-generating conceptual framework integrating PsyCap-related psychological resources within cancer-related dyadic processes. The framework represents a conceptual synthesis of the available literature rather than an empirically validated or evidence-derived causal model. Directional arrows depicting stress-amplifying, stress-buffering, and bidirectional dyadic processes are intended to represent hypothesized relationships and should not be interpreted as established causal pathways.

### 3.2 Conceptualization of PsyCap in cancer care

Across the included literature, PsyCap was commonly conceptualized as comprising self-efficacy, optimism, hope and resilience (Luthans and Youssef, 2004; Luthans et al., 2005; Zhang et al., 2010; Luthans et al., 2006; Luthans et al., 2010). In oncology settings, these components have been conceptualized in relation to illness management, treatment experiences and adaptation to cancer. Across the literature, self-efficacy has been described in relation to symptom management, treatment adherence and communication with healthcare professionals. Optimism has generally been conceptualized as positive expectations regarding future outcomes. Hope has been linked to goal-directed thinking and the identification of pathways for coping with illness-related challenges. Resilience has been described as the capacity to adapt to adversity associated with diagnosis, treatment and survivorship.

Several studies have examined these components individually. Resilience has been associated with quality of life, distress and survivorship outcomes (MacDonald et al., 2021; Gao et al., 2019; Harms et al., 2019). Hope has been linked to coping, existential adjustment and communication processes (Lin and Tsay, 2005; Feldman and Corn, 2023). Individual PsyCap components, particularly self-efficacy and optimism, have been associated with health-related behaviors and adaptive illness management across cancer and other chronic illness populations (Merluzzi et al., 2019; Millstein et al., 2016).

Taken together, the literature suggests that PsyCap provides an integrative framework that combines several psychological resources commonly examined in psycho-oncology research. Evidence from non-cancer clinical populations further suggests that PsyCap may function as a protective psychological resource that buffers the negative psychological effects of stress (Han et al., 2019). However, studies vary considerably in whether PsyCap is conceptualized as a higher-order construct or as a collection of distinct psychological resources.

### 3.3 Determinants and measurement of PsyCap

#### 3.3.1 Determinants of PsyCap

The current evidence indicates that PsyCap and its resources may diminish after diagnosis and during treatment, but this varies according to cancer type, stage, symptom severity, side-effects of treatment, age, socioeconomic status and social support (Sebri et al., 2021; Presley et al., 2024; Khan et al., 2023; Eala et al., 2022; Yang et al., 2025; Wei et al., 2025).

For patients, pain, fatigue, sleep disturbance, body-image concerns and functional limitations were commonly reported correlates of reduced psychological resources. Uncertainty regarding treatment outcomes and future plans was also frequently associated with lower hope and optimism (Sebri et al., 2021; Presley et al., 2024; Khan et al., 2023; Eala et al., 2022).

For caregivers, caregiving burden, emotional demands, social isolation, anticipatory grief and limited support were frequently reported challenges (Lee et al., 2018; Chua et al., 2020; Wang J. et al., 2024; Gu et al., 2024; Wu Y. T. et al., 2025; Sun et al., 2024). Several studies suggested that caregiver psychological resources may also influence patient adaptation through communication, practical assistance and emotional support (Manne and Badr, 2008; Weitkamp et al., 2021; Wang et al., 2021).

Evidence also indicated that PsyCap may fluctuate across different phases of the cancer trajectory. Diagnosis, active treatment, survivorship and advanced illness appear to present distinct psychological challenges, suggesting that PsyCap is dynamic rather than static.

#### 3.3.2 Measurement of PsyCap

Measurement approaches varied substantially across studies. The Psychological Capital Questionnaire was the most frequently cited measure of PsyCap (Luthans et al., 2005), although it was originally developed in organizational settings. Several studies reported adaptations of PsyCap measures for oncology populations, including cancer-specific instruments developed and validated in China (Zhang et al., 2010; Cui et al., 2021).

Considerable heterogeneity was observed in outcome measurement. Some studies assessed PsyCap as a higher-order construct, whereas others focused on individual components such as hope, resilience, self-efficacy or optimism. Most measures relied on self-report instruments. Caregiver-specific and dyadic measures were relatively uncommon, and cultural adaptations varied across studies (Wang et al., 2021; von Heymann-Horan et al., 2019; Duan et al., 2024).

Overall, the evidence suggests growing recognition of the need for oncology-specific approaches to PsyCap measurement, particularly for caregiver and dyadic research.

### 3.4 Outcomes associated with PsyCap

#### 3.4.1 Patient outcomes

Across the included studies, psychological resources consistent with the PsyCap framework—whether assessed as a composite or as separate components—were associated with better psychological adjustment, coping, self-management, and quality of life among patients with cancer (MacDonald et al., 2021; Harms et al., 2019; Wei et al., 2025). Evidence suggests that these psychological resources may influence how individuals perceive and respond to cancer-related stressors, thereby facilitating adaptation throughout the disease trajectory. Similar mediation effects of PsyCap have also been reported in chronic disease populations, where PsyCap partially explained the relationship between social support and treatment burden (Gu et al., 2021). However, it is important to note that direct evidence concerning composite PsyCap in cancer settings is limited, with the majority of studies focusing instead on individual resources such as resilience and hope.

Several studies reported associations between PsyCap and symptom-related outcomes. Symptom burden, perceived social support and PsyCap were found to be associated with social isolation among patients receiving chemotherapy (Wei et al., 2025). In addition, latent profile analysis identified substantial heterogeneity in PsyCap-related distress among patients with pancreatic cancer, suggesting that psychological resources may contribute to differences in emotional responses to illness (Yang et al., 2025).

Studies examining individual PsyCap components reported similar findings. Resilience was associated with improved quality of life and lower psychological distress (MacDonald et al., 2021; Gao et al., 2019; Harms et al., 2019), whereas hope was linked to coping and existential adjustment (Lin and Tsay, 2005; Feldman and Corn, 2023). These findings are broadly consistent with previous evidence linking individual PsyCap components, particularly self-efficacy and optimism, to health-related behaviors and adaptive illness management across cancer and other chronic illness populations (Merluzzi et al., 2019; Millstein et al., 2016).

Although the direction of causality remains uncertain, the available evidence consistently indicates that PsyCap and its constituent resources are positively associated with a range of psychological and behavioral outcomes in oncology populations.

#### 3.4.2 Caregiver outcomes

Evidence specifically examining caregiver PsyCap was limited. Available studies suggested associations between psychological resources, caregiving experiences and emotional outcomes.

Studies of cancer caregivers identified relationships among social support, resilience, positive caregiving experiences and psychological distress (Sun et al., 2024; Jiang et al., 2022). Several publications also highlighted the importance of positive psychological outcomes as a priority area for caregiver research and intervention development (Gudenkauf et al., 2025; Amonoo et al., 2024).

Although the evidence base remains comparatively small, existing findings consistently suggest that psychological resources play an important role in caregiver adjustment.

#### 3.4.3 Dyadic outcomes

A smaller body of literature examined PsyCap-related processes at the dyadic or family level. Studies of advanced cancer populations reported associations between family functioning, resilience and quality of life (Wang et al., 2021).

Dyadic coping theory was frequently used to explain how communication, shared coping and relational processes may influence adjustment among patients and caregivers (Weitkamp et al., 2021; Zemp et al., 2017; Gao et al., 2025; von Heymann-Horan et al., 2019). Collectively, these studies indicate that PsyCap-related processes may operate within interpersonal systems rather than solely at the individual level.

### 3.5 Intervention strategies targeting PsyCap-related psychological resources

#### 3.5.1 Mindfulness-based interventions

Mindfulness-based interventions, including mindfulness-based stress reduction, mindfulness-based cognitive therapy and acceptance and commitment therapy (ACT), were among the most frequently studied psychosocial interventions (Williams and Kuyken, 2012; Hayes, 2016; Miller, 2015; Oberoi et al., 2020; Chayadi et al., 2022). Evidence from non-oncology populations also suggests that ACT-based interventions may improve psychological capital, providing a potential mechanism relevant to cancer care (Rava, 2021).

Systematic reviews and meta-analyses reported beneficial effects on anxiety, depression, fatigue, stress and quality of life (Oberoi et al., 2020; Chayadi et al., 2022). Evidence from caregiver populations outside oncology also suggests that mindfulness-based interventions can improve mental health outcomes and reduce caregiving burden, supporting their potential relevance for cancer caregivers (Saragih et al., 2024). Internet-based mindfulness interventions also demonstrated positive effects on psychological outcomes and perceived stress (Wang T. et al., 2024). One randomized trial suggested the feasibility of mindfulness-based approaches for patients with advanced cancer and their caregivers (Mosher et al., 2024).

Mindfulness-based interventions represent one of the most extensively studied psychosocial approaches in oncology. However, most studies focused on distress reduction rather than direct assessment of PsyCap. The intervention content and dose, duration of follow-up, use of self-reported outcomes, lack of trials specific to caregivers, and lack of mediation analyses are major limitations. Future research should directly measure PsyCap and include mediation analyses of changes in PsyCap on distress, quality of life and coping.

#### 3.5.2 Exercise and body–mind interventions

Exercise and body–mind interventions have been investigated as approaches to improve both physical and psychological outcomes among people with cancer. Laughter yoga demonstrated positive effects on perceived stress, psychological capital and exercise capacity among patients receiving chemotherapy for lung cancer (Han et al., 2023). Broader evidence from non-oncology populations also supports its potential benefits for mental health and well-being (Bressington et al., 2018). Similar approaches have also been explored among terminally ill cancer patients and family caregivers, with reported benefits for mood, pain and burnout (Moon et al., 2024). Traditional body–mind practices such as Baduanjin and Tai Chi have also been evaluated in oncology populations (Kraft et al., 2024; Wu J. et al., 2025). Findings from non-cancer populations further suggest that physical activity may enhance PsyCap through improvements in mastery, self-control and perceived social support (Salvador-Ferrer et al., 2024; Lin et al., 2025).

The results are mixed. Body–mind and exercise interventions are moderately supported for fatigue, mood and quality-of-life outcomes in cancer, but lack direct evidence for PsyCap (often in small, pilot studies) (Han et al., 2023; Moon et al., 2024; Wu J. et al., 2025). Dose, intensity, safety, adherence and timing of interventions is variable. Exercise programs for caregivers and couples are not as well developed, and may build a routine and support for the patient.

#### 3.5.3 PERMA-informed positive psychological interventions

PERMA-informed positive psychological interventions have emerged as a promising strategy for enhancing psychological well-being and PsyCap-related resources in cancer care (Donaldson et al., 2022; Lorenz et al., 2023; Grosvenor et al., 2023). Grounded in the PERMA framework (Positive Emotion, Engagement, Relationships, Meaning, and Accomplishment), these interventions aim to cultivate positive psychological strengths through activities such as gratitude exercises, strengths identification, meaning-making, goal setting and relationship enhancement. Given their focus on hope, optimism, resilience and personal growth, they may provide an important pathway for strengthening PsyCap.

Evidence from the reviewed studies suggests beneficial effects across a range of cancer populations. PERMA-based interventions improved psychological well-being, emotional adjustment and quality of life among patients with breast cancer and acute myeloid leukemia (Xie and Huang, 2025; Fang et al., 2023; Wang D. et al., 2024). A web-based positive psychology program was also associated with improvements in psychological capital, emotional status and quality of life among patients receiving chemotherapy for gastric cancer (Xin and Zhao, 2024). Beyond patient-focused interventions, family-centered and caregiver-focused positive psychology programs demonstrated improvements in psychological health, quality of life and positive adaptation among both patients and caregivers (Amonoo et al., 2024; Zhang et al., 2023). Overall, these findings suggest that PERMA-informed interventions may be a feasible approach for promoting PsyCap-related resources in cancer care. However, most studies assessed quality of life and psychological well-being rather than PsyCap directly, and further research is needed to clarify whether improvements in outcomes are mediated through changes in PsyCap.

#### 3.5.4 Dyadic and family-based interventions

Dyadic and family-based interventions are well aligned with the realities of cancer care, where patients and caregivers often face shared psychological and practical challenges. Evidence suggests that couple- and family-focused interventions can improve relationship quality, coping, quality of life, psychological distress and caregiver burden (Gao et al., 2025; von Heymann-Horan et al., 2019; Wang et al., 2025; Sun et al., 2025; Liu et al., 2024; Chen et al., 2023). A palliative care trial further demonstrated that dyadic coping processes can be strengthened through intervention and may contribute to improved psychosocial outcomes (von Heymann-Horan et al., 2019).

Although evidence remains limited, findings suggest that interventions involving both patients and caregivers may achieve benefits beyond those of individual-focused approaches. However, existing studies are often small, heterogeneous and focused primarily on spouse or partner dyads. Family-focused interventions may be particularly important in cultures where caregiving responsibilities are shared among adult children, siblings and multigenerational family members, yet these populations remain underrepresented in the literature (Zhang et al., 2023; Broad and Luthans, 2020).

#### 3.5.5 Digital and stepped-care approaches

Hybrid and digital approaches can overcome difficulties in scalability, travel, fatigue and time pressures on carers. Internet-based mindfulness-based interventions have proven feasible in oncology (Wang T. et al., 2024), and positive psychology or dyadic support modules can be delivered online (Xin and Zhao, 2024).

Hybrid and digital approaches may address common barriers such as travel burden, fatigue and time pressures. Internet-based mindfulness interventions have demonstrated feasibility in oncology settings (Wang T. et al., 2024), and positive psychology or dyadic support modules can be delivered online (Xin and Zhao, 2024). A stepped-care logic has been proposed in this context, whereby less intensive self-guided modules may suit individuals with relatively preserved psychological resources, whereas more intensive therapist-supported formats may be indicated for dyads experiencing elevated distress or caregiving strain.

## 4 Discussion

### 4.1 Main findings

Building upon the integrative framework presented in Figure 1, three overarching findings emerged regarding psychological capital in cancer patients, family caregivers and patient–caregiver dyads. This figure is presented as a hypothesis-generating synthesis rather than an empirically validated model, particularly because direct evidence concerning dyadic PsyCap remains limited.

First, the literature consistently highlighted positive associations between psychological resources—encompassing both composite PsyCap and its individual components and psychosocial outcomes. However, relatively few oncology studies directly assessed PsyCap as a higher-order construct; instead, much of the available evidence focused on individual PsyCap components, particularly resilience, hope, optimism, and self-efficacy. This distinction is important when interpreting the evidence, as findings concerning individual components should not be generalized to composite PsyCap (MacDonald et al., 2021; Harms et al., 2019; Wei et al., 2025). Collectively, these findings support the relevance of positive psychological resources within the broader PsyCap framework. Similar patterns were observed among caregivers, where resilience and positive caregiving experiences were associated with lower psychological distress (Jiang et al., 2022).

Second, evidence relating to PsyCap and its component resources suggested that psychological resources are closely associated with the clinical and caregiving context, with symptom burden, financial strain, treatment demands and caregiving responsibilities linked to lower levels of both composite PsyCap and its individual component resources such as resilience (Khan et al., 2023; Wei et al., 2025; Gu et al., 2024; Wu Y. T. et al., 2025; Sun et al., 2024). These findings suggest that psychological resources fluctuate across the cancer trajectory rather than remaining stable over time.

Third, intervention studies suggested that psychological resources relevant to PsyCap may be strengthened through structured psychosocial interventions. Mindfulness-based approaches, positive psychology programs and dyadic interventions were associated with improvements in psychological well-being, coping and quality of life across cancer populations (von Heymann-Horan et al., 2019; Wang T. et al., 2024; Xin and Zhao, 2024; Zhang et al., 2023). However, relatively few studies directly measured PsyCap, making it difficult to determine whether these intervention effects were mediated through changes in PsyCap itself.

### 4.2 Interpreting PsyCap in the context of cancer care

One important implication of these findings is that PsyCap may need to be understood differently in cancer care than in its original organizational context. Although PsyCap was initially conceptualized as an individual psychological resource (Luthans and Youssef, 2004; Luthans et al., 2006; Luthans et al., 2010), the evidence reviewed here suggests that its development and expression are closely linked to changing illness demands and interpersonal relationships.

Across the cancer trajectory, patients and caregivers experience evolving physical, emotional and social challenges that may influence the psychological resources underpinning PsyCap, including hope, resilience, optimism and self-efficacy, over time (Khan et al., 2023; Wei et al., 2025; Gu et al., 2024; Wu Y. T. et al., 2025; Sun et al., 2024). Rather than representing fixed personal characteristics, these psychological resources appear responsive to disease progression, symptom burden, treatment experiences and available social support. This dynamic perspective may help explain why individuals with similar clinical characteristics often differ substantially in their psychological adjustment and quality of life.

At the same time, the review indicates that many psychological resources relevant to PsyCap operate within interpersonal contexts. Studies of dyadic coping, communication and family functioning consistently showed that patient and caregiver adaptation is shaped by reciprocal interactions rather than by individual psychological processes alone (Manne and Badr, 2008; Weitkamp et al., 2021; Wang et al., 2021; Zemp et al., 2017; Gao et al., 2025; von Heymann-Horan et al., 2019; Wang et al., 2025; Sun et al., 2025). Although relatively few studies directly examined PsyCap as a higher-order construct, findings from studies investigating its component resources provide preliminary and indirect support for the proposition that PsyCap may extend beyond the individual and operate within patient–caregiver relationships.

Taken together, these observations support viewing PsyCap as a useful conceptual framework for understanding psychological adaptation in cancer care, while highlighting the need for future studies to examine whether PsyCap itself operates as a dynamic and relational construct across the cancer trajectory.

### 4.3 Implications for intervention development and supportive oncology practice

The findings of this review support a growing shift in psycho-oncology from a predominantly deficit-oriented perspective toward a strengths-based approach to cancer adaptation. Rather than focusing solely on the reduction of distress, PsyCap highlights psychological resources that may help individuals maintain functioning and adapt to illness-related challenges. Importantly, self-efficacy, optimism, hope and resilience should not be viewed as forms of unrealistic positivity. Instead, these resources may coexist with fear, uncertainty and symptom burden while facilitating more adaptive coping and adjustment throughout the cancer trajectory (Lin and Tsay, 2005; Feldman and Corn, 2023).

The review suggests that psychological resources captured by the PsyCap framework—whether composite or component-level—may represent potentially modifiable targets for supportive oncology care. However, direct evidence for changes in composite PsyCap remains limited. Existing intervention studies indicate that psychological resources can be strengthened through a variety of approaches, including mindfulness-based programs, PERMA-informed positive psychological interventions, exercise and body–mind practices, and dyadic or family-centered interventions (Wang T. et al., 2024; Mosher et al., 2024; Han et al., 2023; Moon et al., 2024; Xie and Huang, 2025; Fang et al., 2023; Wang D. et al., 2024; Xin and Zhao, 2024; Zhang et al., 2023). Evidence from non-oncology populations further suggests that some of these approaches may promote well-being and adaptive psychological functioning across different health and community settings, providing additional support for their application in cancer care (Kovich et al., 2023; Luo et al., 2025). Although most interventions were not explicitly designed to increase PsyCap, many appear to influence one or more of its core dimensions, particularly hope, resilience and self-efficacy. Building on this empirical pattern, we propose that PsyCap may function as a unifying framework through which diverse psychosocial interventions exert their effects. For example, exercise and mind–body practices (e.g., Tai Chi, Baduanjin) may bolster self-efficacy through mastery experiences and symptom management, while also enhancing resilience via improved physiological stress regulation (Salvador-Ferrer et al., 2024; Lin et al., 2025). Similarly, mindfulness and PERMA-informed interventions may cultivate optimism and hope by fostering positive reappraisal and goal-setting. This multi-pathway model provides a theoretical rationale for why interventions grounded in different therapeutic traditions can converge on similar improvements in psychological adaptation and quality of life. Conceptually, PsyCap may therefore provide a useful integrative lens for understanding and designing supportive care interventions for patients and caregivers across the cancer trajectory.

Another important implication is the need to extend supportive care beyond individual patients, as cancer-related adaptation occurs within interpersonal and family contexts where psychological resources may influence both patients and caregivers (Wang et al., 2021; von Heymann-Horan et al., 2019; Jiang et al., 2022; Li et al., 2022). Interventions that strengthen communication, shared coping and family functioning may therefore offer advantages over approaches focused solely on individual distress management. This perspective may be particularly relevant in cultures where family members play a central role in caregiving and treatment decision-making (Zhang et al., 2023; Broad and Luthans, 2020).

Taken together, these findings suggest that PsyCap may offer a valuable conceptual framework for understanding adaptation and guiding supportive care efforts in cancer populations. Adopting such a framework could usefully complement traditional deficit-focused approaches by directing attention toward psychological resources that support long-term adjustment and quality of life, alongside the management of distress.

### 4.4 Limitations of the current evidence, limitations of the review, and future directions

Although the evidence base on PsyCap in oncology has expanded in recent years, important methodological limitations remain. To distinguish between limitations of the existing literature and those of the present review, these issues are considered separately before outlining priorities for future research.

#### 4.4.1 limitations in the current literature

The reviewed literature demonstrated several recurring methodological limitations. First, conceptual inconsistency remains a major challenge. While some studies conceptualized PsyCap as a higher-order construct, others examined only individual components such as resilience, hope, optimism or self-efficacy (Luthans and Youssef, 2004; Luthans et al., 2005; Zhang et al., 2010; Luthans et al., 2006; Luthans et al., 2010). This variability complicates comparisons across studies and makes it difficult to determine whether observed findings reflect PsyCap as an integrated construct or only specific psychological resources. Second, measurement remains underdeveloped within oncology. Although the Psychological Capital Questionnaire is the most frequently used measure of PsyCap, it was originally developed in occupational settings and may not fully capture the experiences of cancer patients and family caregivers (Luthans et al., 2005). Oncology-specific instruments have begun to emerge (Cui et al., 2021), but validated measures for caregivers and patient–caregiver dyads remain limited. Third, the intervention evidence remains relatively limited. Many studies involved small samples, single-center designs, short follow-up periods and heterogeneous outcome measures (Amonoo et al., 2024; Wang T. et al., 2024; Mosher et al., 2024; Han et al., 2023; Moon et al., 2024; Xie and Huang, 2025; Fang et al., 2023). Moreover, relatively few intervention studies directly assessed PsyCap as a primary outcome or explicitly examined whether changes in PsyCap mediated improvements in psychological or clinical outcomes. Consequently, the mechanisms through which interventions may influence PsyCap remain insufficiently understood.

#### 4.4.2 Limitations of this review

Several limitations of this review should also be acknowledged. The included publications were heterogeneous in study design and publication type, and no study-design filter was applied during eligibility assessment, which limited direct comparison across evidence types and precluded quantitative synthesis. No formal risk-of-bias or methodological quality appraisal was undertaken, and therefore the methodological strength of individual publications could not be systematically differentiated. Third, publication-level search provenance was not prospectively recorded, so the exact contribution of database searching, citation tracking, and purposive searching could not be retrospectively quantified. Although these approaches served distinct purposes and were explicitly described in the Methods and Results, the lack of publication-level provenance limits the ability to report mutually exclusive source counts. Furthermore, thematic synthesis and evidence mapping involved a degree of interpretive judgment. To enhance consistency, predefined thematic domains and explicit evidence-mapping procedures were applied throughout the review, although alternative thematic organizations may also be reasonable. The predefined eligibility criteria, including language and publication-type restrictions, may also have limited the breadth of the evidence identified and introduced the possibility of language or publication bias. Finally, the available evidence was dominated by cross-sectional studies, limiting conclusions regarding causal relationships and changes in PsyCap over time. Evidence relating to caregivers, patient–caregiver dyads and family systems also remains comparatively limited, and this review focused primarily on patients and family caregivers rather than healthcare professionals or organizational factors that may also influence psychological resources in oncology.

#### 4.4.3 Future directions

Addressing these gaps warrants several priorities: (a) conceptual and measurement refinement, including oncology-specific and dyadic tools; (b) adequately powered RCTs that explicitly test PsyCap as a mediator; (c) longitudinal designs tracking PsyCap trajectories across the cancer continuum; (d) dyadic analytical approaches such as actor–partner interdependence models; and (e) implementation research evaluating feasibility, scalability and sustainability in routine practice. Addressing these priorities will strengthen both the evidence base and the translation of PsyCap into supportive oncology practice. Future studies should also distinguish more clearly between PsyCap as a higher-order construct and its individual components, allowing stronger conclusions regarding the mechanisms through which PsyCap contributes to cancer adaptation.

## 5 Conclusion

This review of PsyCap in cancer patients and caregivers across determinants, measurement, outcomes and interventions yielded three main conclusions.

First, PsyCap—conceptualized as a higher-order composite of self-efficacy, optimism, hope and resilience—offers an integrative, strengths-based framework for understanding adaptation in cancer care. Across studies, PsyCap and its components were consistently associated with better psychological adjustment, coping, self-management and quality of life in both patients and caregivers. Emerging evidence also suggests these resources operate at relational levels within patient–caregiver and family systems.

Second, existing interventions show potential for improving psychosocial outcomes and individual PsyCap-related psychological resources. Mindfulness-based programs, PERMA-informed positive psychology interventions, exercise and mind–body practices, and dyadic or family-centered approaches have demonstrated benefits across relevant psychological and psychosocial outcomes. However, most interventions did not directly assess composite PsyCap, and evidence remains insufficient to determine whether composite PsyCap itself can be modified through intervention.

Third, future research should prioritize conceptual clarity (e.g., whether PsyCap offers incremental value over its components), oncology-specific and dyadic measurement tools, longitudinal designs, intervention trials with PsyCap as a primary outcome and mediation tests, and implementation research in real-world settings.

Addressing these priorities will clarify how, when and for whom PsyCap can be most effectively cultivated. With such evidence, PsyCap could become a valuable framework for precision supportive oncology—helping clinicians not only alleviate distress but also strengthen the resources that enable patients and families to adapt and maintain quality of life.
