# Supplementary material for: Psychological capital in cancer patients and family caregivers: determinants, measurement, outcomes, and intervention strategies
Source: Front Psychol. 2026 Sep 23;17:1957751. doi: 10.3389/fpsyg.2026.1957751 (PMC13643844; doi:10.3389/fpsyg.2026.1957751)
Supplement: Supplementary file 1 [file Supplementary_file_1.docx]

**Appendix**

**Table S1. Representative Database Search Framework Used for Literature Identification**

| **Database** | **Database-specific search strategy** |
| --- | --- |
| PubMed/MEDLINE | ("Neoplasms"[Mesh] OR cancer*[tiab] OR neoplasm*[tiab] OR oncolog*[tiab] OR tumor*[tiab] OR tumour*[tiab] OR chemotherapy[tiab] OR survivorship[tiab] OR "palliative care"[tiab]) AND ("psychological capital"[tiab] OR PsyCap[tiab] OR "positive psychological capital"[tiab] OR self-efficacy[tiab] OR "self efficacy"[tiab] OR optimism[tiab] OR hope[tiab] OR resilience[tiab]) AND (patient*[tiab] OR caregiver*[tiab] OR carer*[tiab] OR spouse*[tiab] OR partner*[tiab] OR dyad*[tiab] OR famil*[tiab]). Intervention-focused supplementary search: add AND (intervention*[tiab] OR program*[tiab] OR therap*[tiab] OR mindfulness[tiab] OR exercise[tiab] OR yoga[tiab] OR "Tai Chi"[tiab] OR Baduanjin[tiab] OR laughter[tiab] OR "positive psychology"[tiab] OR dyadic[tiab] OR family-centered[tiab] OR family-centred[tiab] OR web-based[tiab] OR digital[tiab]). |
| Web of Science Core Collection | TS=(cancer* OR neoplasm* OR oncolog* OR tumor* OR tumour* OR chemotherapy OR survivorship OR "palliative care") AND TS=("psychological capital" OR PsyCap OR "positive psychological capital" OR self-efficacy OR optimism OR hope OR resilience) AND TS=(patient* OR caregiver* OR carer* OR spouse* OR partner* OR dyad* OR famil*). Intervention-focused supplementary search: add AND TS=(intervention* OR program* OR therap* OR mindfulness OR exercise OR yoga OR "Tai Chi" OR Baduanjin OR laughter OR "positive psychology" OR dyadic OR "family-centered" OR "family-centred" OR "web-based" OR digital). |
| CINAHL (EBSCOhost) | [(MH "Neoplasms+") OR TI/AB (cancer* OR neoplasm* OR oncolog* OR tumor* OR tumour*)] AND [TI/AB ("psychological capital" OR PsyCap OR "positive psychological capital" OR self-efficacy OR optimism OR hope OR resilience)] AND [TI/AB (patient* OR caregiver* OR carer* OR spouse* OR partner* OR dyad* OR famil*)]. The intervention-focused search added intervention, program, therapy, mindfulness, exercise, yoga, Tai Chi, Baduanjin, laughter, positive psychology, dyadic, family-centered/family-centred, web-based, and digital terms. |
| PsycINFO (EBSCOhost) | [(DE "Cancer") OR TI/AB (cancer* OR neoplasm* OR oncolog* OR tumor* OR tumour*)] AND TI/AB ("psychological capital" OR PsyCap OR "positive psychological capital" OR self-efficacy OR optimism OR hope OR resilience) AND TI/AB (patient* OR caregiver* OR carer* OR spouse* OR partner* OR dyad* OR famil*). The intervention-focused search added intervention, program, therapy, mindfulness, exercise, yoga, Tai Chi, Baduanjin, laughter, positive psychology, dyadic, family-centered/family-centred, web-based, and digital terms. |
